# Anti-Thrombotic Activities of Veratramine via Inhibiting Platelet Aggregation and FIIa/FXa

**DOI:** 10.3390/biology15060462

**Published:** 2026-03-13

**Authors:** Gyuri Han, Ga Eun Kim, Dong Ho Park, Jong-Sup Bae

**Affiliations:** 1 Research Institute of Pharmaceutical Sciences, Cell & Matrix Research Institute (CMRI), College of Pharmacy, Kyungpook National University, Daegu 41566, Republic of Korea; 2 Department of Ophthalmology, School of Medicine, Kyungpook National University Hospital, Kyungpook National University, Daegu 41944, Republic of Korea

**Keywords:** veratramine, coagulation cascade, fibrinolysis, endothelium

## Abstract

Background: There is growing interest in plant-derived compounds for managing vascular diseases. Veratramine (VRT), a steroidal alkaloid isolated from plants of the Veratrum genus, exhibits diverse biological effects such as antihypertensive, analgesic, and antitumor activities, yet its influence on hemostasis and thrombus formation has not been characterized. This investigation sought to determine whether VRT exerts anticoagulant effects using integrated in vitro and murine models. Methods: VRT’s anticoagulant profile was comprehensively evaluated using integrated biochemical, cellular, and murine models, including clotting time assays (aPTT/PT), chromogenic enzymatic assays, fibrin polymerization analysis, platelet aggregometry, and endothelial modulation of PAI-1/t-PA under inflammatory conditions. Results: VRT treatment significantly prolonged both intrinsic and extrinsic coagulation times, directly inhibited enzymatic activities of thrombin and FXa, and attenuated their generation by endothelial cells. Additionally, VRT interfered with fibrin clot formation and diminished agonist-induced platelet aggregation. Ex vivo coagulation analyses confirmed its anticoagulant action, while endothelial studies revealed a reduced PAI-1/t-PA ratio following VRT exposure. Conclusions: These data establish VRT as possessing novel direct dual inhibition of thrombin and FXa alongside suppression of fibrin polymerization, platelet reactivity, and PAI-1 expression—positioning it as a promising multifunctional anticoagulant agent. While preclinical murine models preclude direct clinical translation absent pharmacokinetic data, these findings warrant further mechanistic and translational investigation.

## Simple Summary

Vascular inflammation and blood clotting problems contribute to many serious diseases like sepsis and heart conditions, causing tissue damage and poor outcomes. This study explored whether veratramine, a natural compound from certain lily plants known for other health benefits like lowering blood pressure, could help prevent harmful blood clots. Using lab tests on human blood cells and experiments in mice, we found that veratramine safely slows down key steps in blood clotting, reduces platelet clumping, and supports the body’s natural clot-breaking process, especially when inflammation triggers clotting. Importantly, it works at safe doses without harming cells. These results suggest veratramine could be developed into a new, plant-based medicine to protect blood vessels and treat clotting-related illnesses, potentially offering fewer side effects than current drugs and benefiting patients worldwide by improving outcomes in inflammatory and heart diseases.

## 1. Introduction

The maintenance of vascular integrity relies on hemostasis, a physiological response that seals injured vessels through the formation of a platelet-fibrin clot [1,2]. This process is governed by a precise balance between clot-promoting factors and natural anticoagulant mechanisms that preserve blood fluidity [1,2]. Disruption of this equilibrium, triggered by endothelial damage, inflammatory mediators, circulatory disturbances, or hypercoagulable states, can precipitate pathological thrombus formation [1]. Coagulation proceeds via sequential proteolytic activation of circulating enzyme precursors, culminating in thrombin generation and subsequent fibrin mesh stabilization [2]. Central to this cascade is activated factor X (FXa), which assembles into the prothrombinase complex on phospholipid membranes to catalyze thrombin production from prothrombin [2].

Thrombotic complications are a major determinant of illness burden worldwide, substantially contributing to global morbidity, emphasizing the pressing demand for antithrombotic therapies that offer improved safety and greater target selectivity [3]. Coagulation is initiated when vascular damage exposes blood components to TF-expressing cells, a key event in both arterial and venous thrombosis [2]. This response can be potentiated by inflammatory mediators released following tissue injury or infection, which promote a procoagulant endothelial phenotype. Such activation facilitates leukocyte adhesion via upregulated surface receptors, enabling adherent monocytes to synthesize TF and bind coagulation factors, thereby amplifying thrombotic progression [2,3].

**Figure 1:**
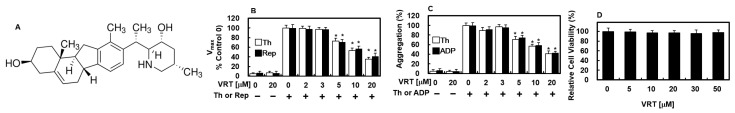
Effects of VRT on fibrin polymerization in human plasma and its cytotoxic profile. (**A**) Chemical structure of VRT. (**B**) Thrombin (Th, white box)- or reptilase (Rep, block box)-mediated fibrin polymerization was assessed in the presence of increasing concentrations of VRT using a turbidimetric method. Data are presented as the maximal polymerization rate (V_max_), normalized to control values and expressed as a percentage. (**C**) Aggregation of mouse platelets stimulated with thrombin (Th, 3 U/mL, white box) or ADP (10 μM, block box) was measured following pretreatment with VRT at various doses. The vehicle control consisted of 0.2% DMSO. (**D**) Cell viability was evaluated using the MTT assay after exposure to VRT. Results are shown as mean ± SD from three independent experiments, each conducted in triplicate. * *p* < 0.01 compared to thrombin alone.

Plant-derived bioactive molecules are increasingly regarded as attractive substitutes for traditional anticoagulant agents because of their favorable safety characteristics and substantial therapeutic promise [4]. Among these, veratramine (VRT, Figure 1A)-steroidal alkaloid found in select members of the Liliaceae family-has drawn attention for its diverse pharmacological effects [5]. Prior investigations have documented its ability to lower blood pressure, block sodium channels, and alleviate pain [6,7]. Furthermore, VRT has been associated with anticancer activity, antihypertensive effects, and cosmetic applications [8,9,10]. However, its effects on the coagulation cascade remain entirely unexplored—no studies have investigated VRT’s impact on thrombin/FXa activity, fibrin formation, platelet function, or fibrinolysis regulation, representing a critical knowledge gap given its structural similarity to bioactive steroidal scaffolds. Notably, Li et al. [11] reported that VRT exhibits significant anti-inflammatory properties. Given the well-established crosstalk between inflammation and coagulation, where inflammatory stimuli promote thrombotic responses and coagulation proteins modulate immune activity, this interplay plays a vital role in host defense mechanisms [12,13]. Building on evidence of VRT’s anti-inflammatory and antioxidant actions [11] and given VRT’s anti-inflammatory properties, we hypothesized that it may attenuate TNF-α-induced endothelial procoagulant responses and examined whether VRT exerts anticoagulant effects. To test this hypothesis, we examined its effects on the generation of thrombin and factor Xa, global coagulation parameters [prothrombin time (PT) and activated partial thromboplastin time (aPTT)], and indices of fibrinolytic activity.

Current first-line therapies for thrombotic disorders primarily rely on direct oral anticoagulants (DOACs) such as rivaroxaban (FXa inhibitor) and dabigatran (direct thrombin inhibitor), which offer predictable pharmacokinetics and reduced monitoring needs compared to vitamin K antagonists [14,15]. However, single-target DOACs leave residual activity in the alternate coagulation arm (e.g., rivaroxaban spares thrombin generation), potentially limiting efficacy in high-thrombotic-risk settings like sepsis or cancer-associated thrombosis [16,17]. A natural compound like VRT exhibiting concurrent direct inhibition of both FXa and thrombin—as preliminarily evidenced herein—could theoretically offer synergistic pathway blockade, broader anticoagulant coverage, and a favorable safety profile derived from its evolutionary optimization in plants. Unlike synthetic DOACs, VRT may also demonstrate enhanced reversibility through natural clearance pathways and lower bleeding risk via tissue-specific modulation. These advantages position VRT as a promising candidate for next-generation anticoagulant development.

## 2. Materials and Methods

This study employed a progressive experimental framework spanning biochemical, cellular, in vivo, and ex vivo models to comprehensively delineate VRT’s anticoagulant mechanisms. Biochemical assays first quantified direct enzymatic inhibition and fibrin dynamics, followed by cellular evaluations of endothelial modulation and platelet function.

### 2.1. Reagents

Reagents and materials were obtained from the following commercial sources: Veratramine (VRT, >98% purity) was purchased from Sigma-Aldrich (St. Louis, MO, USA). Recombinant human tumor necrosis factor-alpha (TNF-α) was supplied by Abnova (Taipei City, Taiwan). A neutralizing antibody against tissue factor was obtained from Santa Cruz Biologics (Santa Cruz, CA, USA). Purified coagulation proteins, including Factors V, VII, VIIa, X, Xa, antithrombin III, prothrombin, and thrombin, were sourced from Haematologic Technologies (Essex Junction, VT, USA). Reagents for aPTT and PT assays were provided by Fisher Diagnostics (Middletown, VA, USA). Chromogenic substrates S-2222 (for FXa) and S-2238 (for thrombin) were obtained from Chromogenix AB (Uppsala, Sweden). The direct factor Xa inhibitor rivaroxaban and the direct thrombin inhibitor argatroban were acquired from Santa Cruz Inc. (Dallas, TX, USA). Enzyme-linked immunosorbent assay (ELISA) kits for quantifying plasminogen activator inhibitor type 1 (PAI-1) and tissue-type plasminogen activator (t-PA) were purchased from American Diagnostica Inc. (Stamford, CT, USA). All other chemicals and reagents used in this study were of analytical grade.

### 2.2. Isolation of Human Plasma

Peripheral venous blood was obtained from ten healthy, fasting donors (six female, four male; age range 24–28 years) following an overnight fast. All participants provided written informed consent and had no history of cardiovascular pathology, allergic conditions, or metabolic abnormalities, nor had they taken any medications prior to sampling. Participants reported abstaining from addictive substances and antioxidant supplementation and adhered to a typical mixed (omnivorous) diet. Blood was collected into citrate-containing tubes (0.32% final concentration) and immediately centrifuged at 2000 rpm for 15 min to isolate plasma for subsequent analyses. This study was conducted after receiving approval from the Institutional Review Board of Kyungpook National University Hospital (KNUH2012-01-010) in Daegu, Republic of Korea.

### 2.3. Anticoagulation Assay

Coagulation times were determined using a Thrombotimer (Behnk Elektronik, Norderstedt, Germany) in accordance with established protocols [18]. For each assay, 90 mL of citrated normal human plasma from independent healthy human donors was incubated with 10 µL of VRT at 37 °C for 1 min before measurement. In the aPTT assay, 100 μL of reagent was introduced and incubated for an additional minute, after which coagulation was triggered by adding 100 μL of 20 mM CaCl_2_, and the time to clot formation was recorded. For PT determination, the plasma-VRT mixture was incubated at 37 °C for one minute, followed by the addition of 200 μL of pre-warmed PT reagent (equilibrated at 37 °C for 10 min) and immediate measurement of clotting time. Results for aPTT were expressed in seconds, whereas PT values were reported in seconds and as international normalized ratio (INR), calculated using the formula: (PT sample/PT control)^ISI^.

### 2.4. Platelet Aggregation Assay

Platelet-rich plasma (PRP) from syngeneic mice was prepared by sequential centrifugation: whole blood was spun at 200× *g* to isolate the platelet layer, then centrifuged again at 500× *g* to pellet the platelets. Platelet concentration was standardized to 1 × 10^9^ cells/mL using a hemocytometer. Isolated platelets were resuspended in HEPES buffer (5 mM HEPES, 136 mM NaCl, 2.7 mM KCl, 0.42 mM NaH_2_PO_4_, 2 mM MgCl_2_, 5.6 mM glucose, 0.1% BSA, pH 7.45) containing 1 mM CaCl_2_. Aggregation assays were conducted as previously described [18]. Washed platelets were incubated with VRT at the indicated concentrations in TBS for 3 min prior to stimulation. Aggregation was induced by adding thrombin (3 U/mL) or ADP (10 µM), and the response was monitored using a Chronolog aggregometer (Havertown, PA, USA).

### 2.5. Thrombin- or Reptilase-Catalyzed Fibrin Polymerization

Fibrin polymerization triggered by thrombin or reptilase was monitored turbidimetrically by measuring absorbance at 360 nm at 6 s intervals for 20 min using a TECAN spectrophotometer (Männedorf, Switzerland) at ambient temperature. Plasma samples—either untreated or pre-incubated with VRT—were diluted 1:3 in TBS (50 mM Tris-buffered saline, pH 7.4), and fibrin polymerization was triggered by adding thrombin or reptilase to achieve a final concentration of 0.5 U/mL. The peak polymerization velocity (V_max_), quantified as ΔmOD/min, was determined from turbidity kinetics using the established protocol of Nowak et al. [19].

### 2.6. Cell Culture

Human umbilical vein endothelial cells (HUVECs) were procured from Cambrex Bio Science (Charles City, IA, USA) and propagated using established protocols [20,21]. In brief, cells were grown in EBM-2 basal medium supplemented with endothelial growth factors (Cambrex Bio Science) at 37 °C under 5% CO_2_ in a humidified incubator until reaching confluence. Experiments utilized HUVECs from passages 3 to 5 only.

### 2.7. Animals and Husbandry

Male C57BL/6 mice (6–7 weeks old, ~27 g body weight) were sourced from Orient Bio Co. (Seongnam, Republic of Korea). Upon receipt, mice were acclimated for 12 days before experimental procedures. Animals were maintained in polycarbonate cages (5 mice per cage) under controlled conditions (20–25 °C, 40–45% humidity, 12 h light/dark cycle), with unrestricted access to standard rodent chow and water. All procedures adhered to institutional animal care standards and received approval from the Kyungpook National University Institutional Animal Care and Use Committee (approval No. KNU2024-13).

### 2.8. Cell Viability Assay

Cellular viability activity in response to VRT treatment was determined by the MTT [3-(4,5-dimethylthiazol-2-yl)-2,5-diphenyltetrazolium bromide] reduction assay. HUVECs were plated in 96-well culture plates at a density of 5 × 10^3^ cells per well and incubated for 24 h to allow attachment. Following medium aspiration, cells were exposed to different concentrations of VRT and incubated for an additional 48 h. Following treatment, the supernatant was discarded, and 100 μL of MTT reagent (1 mg/mL in culture medium) was added to each well, followed by a 4 h incubation at 37 °C. The formazan crystals formed were solubilized by adding 150 μL of dimethyl sulfoxide (DMSO), and absorbance was measured at 540 nm using a microplate reader (Tecan Austria GmbH, Grödig, Austria).

### 2.9. Factor Xa Production on the Surfaces of HUVECs

FXa generation on endothelial surfaces was quantified using a chromogenic assay. HUVEC monolayers cultured in 96-well plates were pretreated with VRT for 10 min prior to stimulation with TNF-α (10 ng/mL) for 6 h under serum-free conditions. After stimulation, cells were incubated with FVIIa (10 nM) for 5 min at 37 °C in assay buffer (buffer A containing 5 mg/mL BSA and 5 mM CaCl_2_), in the presence or absence of a TF-blocking antibody (25 µg/mL). FX was then added to a final concentration of 175 nM, and the reaction proceeded for 15 min before being terminated with EDTA-supplemented buffer A. FXa activity was measured by adding a chromogenic substrate and monitoring absorbance at 405 nm for 2 min. The rate of substrate cleavage was converted to FXa concentration using a standard curve established with purified human FXa.

### 2.10. Thrombin Production on the Surfaces of HUVECs

Thrombin production by endothelial cells was measured according to previously described protocols [22,23]. Briefly, confluent HUVEC monolayers were incubated in 300 μL of 50 mM Tris-HCl buffer containing VRT along with 100 pM factor Va and 1 nM factor Xa for 10 min at 37 °C. Prothrombin was subsequently added to achieve a final concentration of 1 μM, and the mixture was incubated for an additional 10 min. Aliquots (10 μL) were then removed in duplicate and added to 40 μL of 0.5 M EDTA in Tris-buffered saline to terminate the reaction. Thrombin activity was assessed by measuring the cleavage of the chromogenic substrate S-2238 at 405 nm, and the concentration was calculated by interpolation from a standard curve established with known amounts of purified thrombin.

### 2.11. Thrombin Activity Assay

To evaluate the effect of VRT on thrombin activity in the presence of antithrombin III (AT III), the compound was diluted in 50 mM Tris-HCl buffer (pH 7.4) containing 7.5 mM EDTA and 150 mM NaCl, with or without 200 nM AT III. Heparin controls were prepared in physiological saline and combined with AT III (200 nM) before addition to the reaction wells. After 2 min preincubation at 37 °C, 150 µL of thrombin (10 U/mL) was added, and the mixture was incubated for another minute at 37 °C. The chromogenic substrate S-2238 (150 µL, 1.5 mM) was then introduced, and absorbance at 405 nm was monitored continuously for 120 s using a TECAN spectrophotometer (Switzerland).

### 2.12. Factor Xa (FXa) Activity Assay

These experimental procedure for evaluating factor Xa activity was conducted analogously to the thrombin activity assay, with the substitution of FXa (1 U/mL) as the enzyme and the chromogenic substrate S-2222 for detection.

### 2.13. In Vivo Bleeding Time

Bleeding time was measured in C57BL/6 mice using the tail transection model described by Dejana et al. [23,24]. Mice were fasted overnight prior to the procedure. One hour following intravenous administration of VRT, the distal 2 mm segment of the tail was amputated. The time required for bleeding to cease completely was recorded, with a cutoff of 15 min applied for statistical purposes in cases where hemostasis was not achieved within this interval.

### 2.14. Ex Vivo Clotting Time

Following an overnight fast, male C57BL/6 mice were administered VRT intravenously in a vehicle containing 0.5% DMSO. One hour after treatment, arterial blood samples (0.1 mL) were collected into 3.8% sodium citrate anticoagulant at a 1:10 ratio (citrate:blood). Plasma was subsequently isolated for ex vivo measurement of aPTT and PT using the coagulation assays described earlier.

### 2.15. ELISA for PAI-1 and t-PA

Conditioned media collected from HUVEC cultures were analyzed for plasminogen activator inhibitor type 1 (PAI-1) and tissue-type plasminogen activator (t-PA) concentrations using enzyme-linked immunosorbent assay (ELISA) kits obtained from American Diagnostica Inc. (Stamford, CT, USA), following the manufacturer’s protocols.

### 2.16. Statistical Analysis

All quantitative data are presented as the mean ± standard deviation (SD) derived from a minimum of three independent experiments, each performed in duplicate. Normality was assessed via the Shapiro–Wilk test (α = 0.05); normally distributed data underwent parametric analysis, while non-normal data used non-parametric equivalents. For multiple-group comparisons, one-way ANOVA followed by Dunnett’s post hoc test (vs. control) was applied to normally distributed data, non-parametric Kruskal-Wallis test with Dunn’s post hoc was used otherwise. Statistical analyses were conducted using SPSS software (version 14.0, SPSS Science, Chicago, IL, USA), with a *p*-value of less than 0.05 considered to indicate statistical significance.

## 3. Results

### 3.1. Effects of VRT on Clotting Time and Bleeding Times

Incubation of human plasma with VRT resulted in significant alterations to coagulation parameters, as assessed by aPTT and PT assays (Table 1). Although its anticoagulant potency was lower than that of heparin or warfarin, VRT dose-dependently prolonged aPTT and PT at concentrations ≥5 μM. aPTT prolongation signifies inhibition of the intrinsic/common coagulation pathway, whereas PT extension reflects extrinsic/common pathway interference. The concentrations needed to double clotting times were 20.6 μM for aPTT and 17.6 μM for PT, suggesting a potential effect on the common pathway. To provide a more interpretable comparison of relative efficacy, we calculated the fold-prolongation of clotting times at equimolar concentrations (20 μM). At this concentration, VRT prolonged aPTT by approximately 1.99-fold compared to control (from 24.6 s to 48.9 s), whereas heparin and warfarin produced 2.39-fold and 2.49-fold prolongations, respectively. Similarly, for PT, VRT showed a 2.15-fold increase (from 12.2 s to 26.2 s), compared to 2.56-fold for heparin and 2.63-fold for warfarin. These data indicate that, within this in vitro assay system at 20 µM, VRT produces approximately 80–85% of the clotting time prolongation observed with heparin and warfarin. This comparison is limited to equimolar conditions in aPTT/PT assays and does not account for differences in mechanism or pharmacokinetics between these agents. To corroborate these in vitro observations, in vivo hemostatic function was assessed using a tail bleeding model in mice. While this model confirms the anticoagulant activity of VRT in a living system, it measures bleeding tendency rather than thrombus prevention, and, therefore, should be interpreted as evidence of anticoagulant effect rather than definitive anticoagulant efficacy. With an estimated mouse blood volume of 72 mL/kg [25], a typical 27 g animal has ~2 mL total blood. Thus, intravenous VRT doses of 0.03, 0.06, 0.09, 0.15, 0.3, or 0.6 mg/kg produced approximate plasma concentrations of 1, 2, 3, 5, 10, or 20 μM, respectively (note: direct PK validation unavailable). As indicated in Table 1, VRT significantly extended tail bleeding times compared to vehicle controls. Furthermore, ex vivo coagulation assays demonstrated dose-dependent prolongations of both aPTT and PT following VRT administration (Table 2).

### 3.2. Effects of VRT on Thrombin-Catalyzed Platelet Aggregation and Fibrin Polymerization and Cellular Viability

The effect of VRT on fibrin clot formation in human plasma was evaluated by monitoring turbidity at 360 nm, as described in Section 2. As shown in Figure 1B, VRT significantly diminished the maximal rate of fibrin polymerization. All test dilutions were prepared in 50 mM TBS (pH 7.4) to maintain uniform pH, and control experiments confirmed that DMSO alone had no effect on coagulation parameters. To explore whether the anticoagulant effect extended to platelet function, thrombin-induced aggregation assays were performed using murine platelets. VRT dose-dependently suppressed aggregation triggered by 3 U/mL thrombin (Figure 1C). To determine whether the antiplatelet effect of VRT is secondary to thrombin inhibition or reflects direct interference with platelet signaling pathways, we evaluated the effect of VRT on aggregation induced by ADP, a non-thrombin agonist that activates platelets through P2Y_1_ and P2Y_12_ receptors. Data showed that VRT dose-dependently suppressed ADP-induced platelet aggregation (10 μM ADP), with significant inhibition observed at concentrations of 5 μM and above. This inhibition of aggregation triggered by an agonist that acts independently of thrombin signaling indicates that VRT exerts direct effects on platelet activation pathways, rather than its antiplatelet activity being solely attributable to thrombin inhibition. To determine whether the observed reduction in turbidity was due to impaired fibrin polymerization rather than decreased fibrinogen cleavage, reptilase-induced polymerization was examined. VRT also significantly inhibited this process (Figure 1B), indicating direct interference with fibrin assembly. Finally, the cytotoxicity of VRT was assessed in HUVECs using the MTT assay following 24 h exposure. Concentrations up to 50 μM had no adverse effect on cell viability (Figure 1D).

### 3.3. Effects of VRT on the Activities of Thrombin and FXa

**Figure 2:**
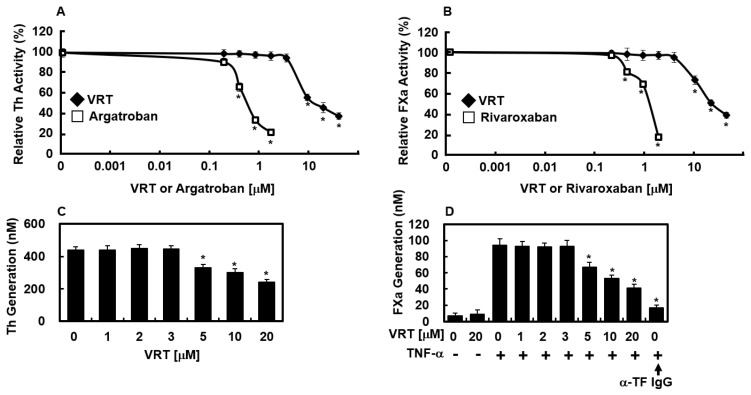
Modulation of thrombin and factor Xa activity and generation by VRT. (**A**) Direct inhibition of thrombin amidolytic activity by VRT was assessed using a chromogenic substrate assay. (**B**) Inhibition of FXa activity by VRT was measured under similar conditions. Argatroban and rivaroxaban served as positive controls for thrombin and FXa inhibition, respectively. (**C**) HUVEC monolayers were incubated with FVa (100 pM) and FXa (1 nM) in the presence of increasing VRT concentrations for 10 min. Prothrombin (1 µM) was then added, and thrombin generation was quantified after 30 min. (**D**) HUVECs were pretreated with VRT for 10 min prior to stimulation with TNF-α (10 ng/mL, 6 h). Following stimulation, cells were exposed to FVIIa (10 nM) and FX (175 nM) with or without anti-TF IgG (25 µg/mL). FXa production was subsequently measured. Data are presented as mean ± SD. * *p* < 0.01 compared to control (**A**–**C**) or TNF-α alone (**D**).

To elucidate the molecular basis of VRT’s anticoagulant action, its inhibitory effects on thrombin and FXa were assessed using chromogenic substrate assays. As illustrated in Figure 2A, VRT reduced thrombin-mediated substrate cleavage in a concentration-dependent manner, indicating direct inhibition of thrombin activity. Argatroban, a well-characterized direct thrombin inhibitor, served as a positive control. Similarly, VRT suppressed FXa activity in a dose-dependent fashion (Figure 2B), with rivaroxaban used as a reference direct FXa inhibitor. These results are consistent with observations from antithrombin assays and suggest that the anticoagulant properties of VRT involve inhibition of two key coagulation proteases, thrombin and FXa, in addition to its effects on fibrin polymerization and interference with both intrinsic and extrinsic pathway components.

### 3.4. Effects of VRT on Production of Thrombin and FXa

Previous work by Sugo et al. established that endothelial cells can support prothrombin activation mediated by FXa [26]. In the present study, incubation of HUVECs with FVa and FXa in the presence of calcium, followed by addition of prothrombin, resulted in thrombin generation (Figure 2C). Treatment with VRT significantly and dose-dependently reduced thrombin formation from prothrombin under these conditions (Figure 2C). Rao and colleagues reported that endothelial surfaces can substitute for procoagulant phospholipids in supporting FX activation [27], while Ghosh et al. demonstrated that FVIIa-mediated FX activation in TNF-α-stimulated HUVECs is TF-dependent [28]. To explore whether VRT interferes with this pathway, FX activation by FVIIa was assessed. Stimulation with TNF-α markedly upregulated TF activity, leading to an approximately 13-fold increase in FX activation compared to unstimulated controls (93.7 ± 4.9 nM vs. 7.1 ± 2.9 nM, Figure 2D). This enhancement was nearly abolished by anti-TF IgG (17.1 ± 3.3 nM). Preincubation with VRT also inhibited FX activation in a concentration-dependent manner (Figure 2D), indicating that VRT effectively suppresses the endothelial generation of both FXa and thrombin.

### 3.5. Effects of VRT on Secretion of PAI-1 or t-PA Protein

**Figure 3:**
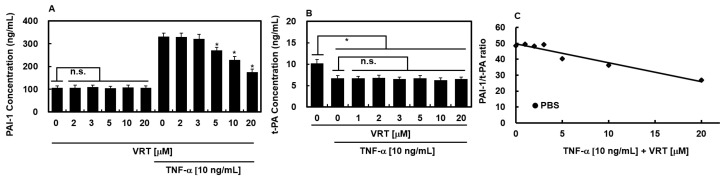
Effects of VRT on PAI-1 and t-PA secretion in HUVECs. (**A**) Cells were treated with increasing concentrations of VRT in the presence or absence of TNF-α (10 ng/mL) for 18 h, after which PAI-1 levels in the culture supernatant were quantified. (**B**) t-PA concentrations were measured in the same conditioned media following VRT and TNF-α treatment under identical conditions. (**C**) The PAI-1/t-PA ratio was calculated from the data shown in (**A**,**B**) to assess net fibrinolytic balance. Values are expressed as mean ± SD. * *p* < 0.01 compared to TNF-α alone or vehicle control; n.s. denotes no statistically significant difference.

TNF-α is known to impair fibrinolytic function in endothelial cells by upregulating PAI-1 expression, thereby altering the t-PA/PAI-1 equilibrium and promoting a prothrombotic state [29,30]. To determine whether VRT modulates this response, HUVECs were incubated with TNF-α in the presence or absence of VRT for 18 h. As shown in Figure 3A, VRT significantly suppressed TNF-α-induced PAI-1 secretion in a concentration-dependent manner, with effects evident at 5 μM and above. Although TNF-α has little effect on t-PA synthesis [31], the net fibrinolytic activity is governed by the ratio of plasminogen activator to its inhibitor [32,33,34]. Consistent with prior observations [35], TNF-α modestly reduced t-PA levels, and this effect was not significantly altered by VRT (Figure 3B). Consequently, TNF-α elevated the PAI-1/t-PA ratio, a shift that was effectively reversed by VRT treatment (Figure 3C).

## 4. Discussion

The present investigation demonstrates that VRT primarily exerts anticoagulant effects by targeting key coagulation proteases. It directly inhibited the amidolytic activity of thrombin and FXa and reduced their generation on endothelial surfaces, thereby attenuating both enzymatic activity and upstream protease production (Figure 2A–D). Given the central roles of FXa in prothrombinase complex formation and thrombin in fibrin generation and platelet activation [2], this dual interference is likely to have a broad impact on thrombus formation across the coagulation cascade. However, our finding that VRT also inhibits ADP-induced platelet aggregation (Figure 1C) reveals an additional, direct effect on platelet signaling pathways independent of thrombin inhibition. This suggests that VRT interferes with common downstream effectors of platelet activation, such as integrin αIIbβ_3_ activation, intracellular calcium mobilization, or granule secretion [36,37,38]. At the final stage of clot formation, VRT directly impairs fibrin polymerization (Figure 1B), as confirmed by reptilase-induced polymerization experiments (Figure 1B), indicating interference with fibrin assembly rather than merely reduced fibrinogen cleavage. Collectively, this multi-tiered mechanism—inhibition of coagulation protease activity, suppression of platelet activation through both indirect (thrombin-dependent) and direct (thrombin-independent) pathways, and disruption of fibrin clot formation—positions VRT as a uniquely multifunctional anticoagulant agent. Furthermore, by reducing the PAI-1/t-PA ratio in inflamed endothelial cells (Figure 3C), VRT may also promote fibrinolytic clearance of formed thrombi, adding a fourth dimension to its anticoagulant profile. This integrated mechanism contrasts with conventional anticoagulants that typically target a single node (e.g., FXa inhibition by rivaroxaban, thrombin inhibition by argatroban, or P2Y_12_ inhibition by clopidogrel), potentially offering advantages in terms of efficacy and safety.

This present investigation demonstrates that VRT exerts significant anticoagulant effects through dual inhibition of critical coagulation proteases, including FXa and thrombin, thereby interfering with both the intrinsic and extrinsic arms of the coagulation cascade. In addition to its enzymatic targets, VRT inhibited fibrin polymerization and platelet aggregation, reinforcing its profile as a versatile anticoagulant agent. These findings position VRT as a promising candidate for therapeutic intervention in thrombotic disorders.

The comparative analysis of VRT with conventional anticoagulants heparin and warfarin reveals that VRT achieves approximately 80–85% of their clotting time prolongation at the same nominal concentration in vitro. However, this equimolar comparison is purely theoretical and assay-based; it does not reflect clinical potency, as heparin and warfarin differ fundamentally from VRT in mechanism of action, pharmacokinetics, and therapeutic use. Therefore, these data should be interpreted only as an exploratory benchmark of relative anticoagulant activity under standardized experimental conditions, not as an indication of therapeutic equivalence. This moderate potency profile may offer certain therapeutic advantages, as agents with excessive anticoagulant activity are associated with increased bleeding risk [39,40]. The relationship between anticoagulant potency and bleeding risk is well-established, with numerous studies demonstrating that the therapeutic window of anticoagulants is determined by the balance between efficacy and safety [41,42]. The intermediate potency of VRT, combined with its multifunctional mechanism targeting both coagulation proteases and platelet aggregation, suggests it may possess a favorable safety profile worthy of further investigation.

VRT also improved endothelial fibrinolytic balance by reversing TNF-α–induced elevation of the PAI-1/t-PA ratio in HUVECs, largely through suppression of PAI-1 secretion without substantially altering t-PA levels (Figure 3A–C). This shift toward a more favorable PAI-1/t-PA profile suggests that VRT not only limits clot formation but may also facilitate fibrin clearance in inflammatory settings.

A substantial body of evidence underscores the bidirectional relationship between inflammation and coagulation, wherein each system modulates the activity of the other [12]. This crosstalk is manifested in processes such as platelet activation, fibrin turnover, and the regulation of natural anticoagulant pathways [12]. The current findings suggest that VRT may attenuate thrombotic responses, potentially contributing to anti-inflammatory effects, though this hypothesis warrants confirmation through well-designed clinical studies. Over the past several decades, conventional anticoagulants such as heparin and coumarin derivatives have remained central to thrombosis management, despite the emergence of newer agents [39,40,43]. Although these drugs are effective, readily available, and can be reversed when necessary, their use requires careful laboratory monitoring due to narrow therapeutic windows and bleeding risks. If VRT is pursued as a candidate anticoagulant, similar considerations regarding monitoring and the potential for thrombotic complications would need to be addressed during its development.

The observation that VRT reduces TNF-α-induced PAI-1 secretion in HUVECs (Figure 3A) is a compelling finding that links its anticoagulant properties to modulation of the inflammatory response. TNF-α is known to upregulate PAI-1 expression through multiple intracellular signaling pathways, including NF-κB, MAPK (ERK, p38, JNK), and PI3K/Akt [31,44]. The NF-κB pathway, in particular, plays a central role in TNF-α-mediated inflammatory responses and has been implicated in PAI-1 transcriptional regulation [31]. Previous studies have demonstrated that various natural compounds exert anti-inflammatory effects through inhibition of NF-κB activation [45]. Given that VRT has been reported to possess anti-inflammatory properties [11], it is plausible that its suppression of PAI-1 secretion may occur through modulation of NF-κB or related signaling cascades. However, the current study was designed to establish the anticoagulant profile of VRT, with the PAI-1/t-PA data serving to demonstrate that VRT can reverse the prothrombotic shift in fibrinolysis associated with inflammation. The precise molecular mechanism by which VRT attenuates PAI-1 expression—whether through NF-κB, MAPK, or other pathways—remains to be elucidated. Future studies employing Western blot analysis of phosphorylated signaling proteins, electrophoretic mobility shift assays for NF-κB DNA binding, nuclear translocation studies, and specific pathway inhibitors will be necessary to definitively identify the signaling pathways modulated by VRT. Such mechanistic investigations represent an important direction for future research as VRT advances toward preclinical development.

An important observation from our study is the differential effect of VRT on the two key regulators of fibrinolysis: while VRT significantly reduced TNF-α-induced PAI-1 secretion in a concentration-dependent manner (Figure 3A), it did not significantly alter t-PA levels (Figure 3B). This selective modulation—targeting the inhibitor rather than the activator—may offer distinct therapeutic advantages in the context of thrombotic disorders. The fibrinolytic system is delicately balanced, and excessive enhancement of plasminogen activator activity (e.g., by increasing t-PA) could theoretically predispose to bleeding complications, particularly in vulnerable patients [46]. In contrast, reducing PAI-1 levels restores endogenous fibrinolytic activity without supra-physiologically amplifying activator function [47]. This mechanistic distinction is clinically relevant: therapeutic strategies that directly increase t-PA (such as recombinant t-PA for acute ischemic stroke) are associated with significant bleeding risk and require careful patient selection and monitoring [42]. By contrast, PAI-1 inhibition has been proposed as a safer alternative that enhances fibrinolysis more physiologically by removing the ‘brake’ rather than pressing the ‘accelerator’ [48]. Furthermore, elevated PAI-1 levels are independently associated with thrombotic risk in various pathological conditions, including sepsis, atherosclerosis, and metabolic syndrome [44]. Therefore, selective reduction of PAI-1 by VRT, without perturbation of t-PA levels, may represent a particularly attractive approach for restoring fibrinolytic balance in prothrombotic, pro-inflammatory states. This selective modulation aligns with the growing interest in developing PAI-1 inhibitors as therapeutic agents [44] and suggests that VRT, through its PAI-1-lowering effect, may offer a safer alternative to direct fibrinolytic activators.

An important question is whether the observed reduction in PAI-1/t-PA ratio (from approximately 4.2 to 2.1 at 20 μM VRT; Figure 3C) is sufficient to meaningfully alter fibrinolytic activity in vivo. Multiple lines of evidence suggest this magnitude is biologically significant. First, mathematical modeling demonstrates that PAI-1 critically controls thrombolysis propagation even at concentrations ten-fold lower than t-PA, indicating that modest PAI-1 reductions can have amplified effects on fibrinolytic efficiency [49]. Second, studies using experimental thrombosis models show that pharmacological PAI-1 inhibition reduces thrombus weight by 20–45% without prolonging bleeding time [50]. Third, in a stasis-induced deep vein thrombosis model, all resolving thrombi (age ≥10 days) exhibited t-PA/PAI-1 ratios >0.2 [51]; VRT increased this ratio from approximately 0.24 to 0.48, exceeding this threshold. Collectively, these data indicate that the approximate 2-fold reduction in PAI-1/t-PA ratio achieved by VRT falls within a range associated with enhanced fibrinolysis and reduced thrombosis in multiple experimental systems. Thus, we propose this modulation is likely sufficient to exert meaningful effects on fibrinolytic flux in vivo, although direct confirmation using functional assays remains an important future direction.

The development of VRT as an anticoagulant presents several potential benefits compared to conventional synthetic agents. To begin with, the production cost of VRT may be substantially lower, as botanical agents typically require less extensive investment in development, clinical trials, and marketing than standard pharmaceuticals. Additionally, preclinical evaluation of VRT’s anticoagulant activity can be reliably conducted using standardized and widely accepted coagulation assays—including PT, aPTT, fibrin polymerization, and platelet aggregation tests—which have been extensively validated for assessing the therapeutic potential of novel anticoagulant candidates [18,41,42,52,53]. The development of VRT as an anticoagulant presents several potential advantages, although these must be considered alongside the need for rigorous safety evaluation. First, natural products have historically served as valuable sources of lead compounds for drug discovery, with approximately one-third of approved drugs between 1981 and 2019 being either natural products or their derivatives [54]. Second, the structural diversity of natural compounds can provide unique pharmacological scaffolds that differ from synthetic small molecules [55]. However, it is important to emphasize that natural origin does not inherently confer safety. Many plant-derived alkaloids—including Veratrum alkaloids themselves—possess significant toxicity, with documented adverse effects ranging from hypotension and bradycardia to neurological symptoms [6,7]. Indeed, Veratrum species have historically been recognized as toxic plants, and their alkaloids can cause severe poisoning [41]. Therefore, any assumption of safety must be rigorously tested through comprehensive toxicological evaluation, including cytotoxicity assays (such as the MTT data presented in Figure 1C), genotoxicity studies, and in vivo toxicity testing. The current study provides initial evidence that VRT lacks cytotoxicity in endothelial cells at concentrations up to 50 μM, but this represents only the first step in a comprehensive safety assessment. Future studies must include detailed toxicological profiling before any therapeutic application can be considered.

Several critical knowledge gaps must be addressed before clinical translation of VRT can be contemplated. Bioavailability and pharmacokinetics: As a steroidal alkaloid, VRT’s physicochemical properties may influence its absorption and metabolism, with many alkaloids exhibiting limited oral bioavailability due to first-pass hepatic metabolism [56]. Pharmacokinetic studies using LC-MS/MS are needed to determine whether VRT achieves and maintains plasma concentrations within the active range identified (5–20 μM) following clinically feasible routes of administration [57]. Therapeutic window and safety: the MTT data provide an initial safety margin of 2.5–10 times relative to active concentrations. However, comprehensive toxicological evaluation—including genotoxicity testing, hERG channel screening, and in vivo toxicity studies—is essential [58]. Given that Veratrum alkaloids are known to cause hypotension, bradycardia, and neurological symptoms at toxic doses [6,7,41], careful dose-escalation studies will be critical. Reversibility and drug interactions: No reversal agent currently exists for VRT, unlike vitamin K antagonists or direct oral anticoagulants, which have specific antidotes [59]. Strategies for managing bleeding complications would need development. Additionally, VRT’s metabolism by cytochrome P450 enzymes has not been characterized, leaving open the possibility of pharmacokinetic interactions with standard anticoagulants or antiplatelet agents [60]. Clinical context: Despite these knowledge gaps, VRT’s multifunctional mechanism—targeting coagulation proteases, platelet activation, and PAI-1—may offer advantages in conditions where inflammation and coagulation are intimately linked, such as sepsis-associated coagulopathy [34]. However, we emphasize that the present findings represent early-stage discovery, and extensive preclinical development is required before clinical translation can be considered.

While the 1 h post-administration time point effectively captured VRT’s anticoagulant effects, future studies incorporating full Pharmacokinetic analysis would further elucidate its absorption, distribution, metabolism, and elimination profile to optimize dosing regimens. All in vivo studies utilized male mice to eliminate estrous cycle variability; however, sex differences in coagulation and pharmacokinetics warrant future investigations in both sexes to ensure translatability. Limitations include theoretical in vivo dosing (extrapolated from blood volume without PK confirmation); future studies will incorporate LC-MS/MS plasma analytics to validate systemic exposure. In addition, the absence of a direct in vivo thrombosis model, such as FeCl_3_-induced arterial or venous thrombosis. The tail bleeding model employed herein demonstrates that VRT exerts anticoagulant effects in vivo, as evidenced by prolonged bleeding time. However, this model assesses hemostatic function and bleeding risk rather than directly measuring protection against pathological thrombus formation [24]. While our in vitro data demonstrating inhibition of platelet aggregation, fibrin polymerization, and coagulation protease activity collectively suggest anticoagulant potential, definitive confirmation requires demonstration of efficacy in established thrombosis models. Several well-validated models are available for this purpose, including FeCl_3_-induced carotid artery thrombosis [61], inferior vena cava ligation-induced venous thrombosis [62], and laser-induced cremaster arteriole thrombosis models [63]. Future studies employing such models will be essential to establish whether the anticoagulant properties of VRT translate into protection against occlusive thrombus formation in vivo. Furthermore, while the absence of cytotoxicity in HUVECs at concentrations up to 50 μM over 48 h is reassuring, we acknowledge that this represents an initial safety assessment rather than a comprehensive toxicological profile. The MTT assay was employed primarily to verify that the observed anticoagulant and antiplatelet effects were not confounded by nonspecific cell death. However, a complete safety evaluation would ideally include longer exposure periods, assessment of additional viability markers such as apoptosis (e.g., annexin V staining, caspase activation), and in vivo toxicity studies. Such comprehensive toxicological profiling represents an important direction for future research as VRT advances toward preclinical development. Nevertheless, for the purpose of the current mechanistic investigation, the MTT data provide adequate assurance that the pharmacological effects described herein occur at non-cytotoxic concentrations. Therefore, while the current findings support VRT as a promising anticoagulant candidate, we temper our conclusions regarding its anticoagulant efficacy pending further investigation.

## 5. Conclusions

In conclusion, this preclinical study demonstrates that VRT exerts anticoagulant effects in vitro and ex vivo by attenuating the generation of FXa and thrombin in endothelial cells, thereby interfering with both the intrinsic and extrinsic coagulation pathways. Furthermore, VRT downregulates PAI-1 secretion induced by TNF-α in cultured endothelial cells, suggesting a potential modulatory role in fibrinolysis. These observations identify VRT as a multifunctional anticoagulant compound in preclinical models, with effects on coagulation proteases, platelet reactivity, and fibrinolytic balance. However, the absence of an in vivo thrombosis model represents a limitation of the current work, and confirmation of true anticoagulant efficacy will require validation in appropriate animal thrombosis models. While these findings provide a scientific rationale for further investigation, we emphasize that they are derived from in vitro and ex vivo experiments, and the translational potential of VRT remains to be established through rigorous in vivo efficacy and safety studies. Future studies should focus on (i) pharmacokinetic profiling to determine oral bioavailability and metabolic stability, (ii) efficacy evaluation in established thrombosis models such as FeCl_3_-induced arterial thrombosis and inferior vena cava ligation, and (iii) structure-activity relationship studies to identify key structural features responsible for its multi-target anticoagulant activity.

## Figures and Tables

**Table 1:** Anticoagulant activity of VRT.

**In Vitro Coagulant Assay**				
Sample	Dose	aPTT (s)	PT (s)	PT (INR)
Control	saline	24.6 ± 0.2	12.2 ± 0.4	1.00
VRT	1 μM	24.7 ± 0.3	12.4 ± 0.4	1.04
	2 μM	24.9 ± 0.5	12.4 ± 0.3	1.04
	3 μM	25.1 ± 0.2	12.5 ± 0.3	1.05
	5 μM	28.5 ± 0.3 *	15.7 ± 0.2 *	1.74 *
	10 μM	35.8 ± 0.3 *	18.9 ± 0.5 *	2.62 *
	20 μM	48.9 ± 0.4 *	26.2 ± 0.3 *	5.37 *
Heparin	20 μM	58.7 ± 0.6 *	31.2 ± 0.5 *	7.89 *
Warfarin	20 μM	61.2 ± 0.9 *	32.1 ± 0.5 *	8.40 *
**In Vivo Bleeding Time**				
Sample	Dose	Tail Bleeding time (s)		*n*
Control	Saline	30.6 ± 0.5		5
VRT	0.03 mg/kg	30.7 ± 0.9		5
	0.06 mg/kg	31.1 ± 0.7		5
	0.09 mg/kg	31.2 ± 0.8		5
	0.15 mg/kg	46.4 ± 1.1 *		5
	0.3 mg/kg	57.4 ± 0.8 *		5
	0.6 mg/kg	75.4 ± 0.9 *		5
Heparin	0.6 mg/kg	81.8 ± 1.2 *		5
Warfarin	0.6 mg/kg	85.7 ± 1.1 *		5

Each value represents the means ± SD (*n* = 5). * *p* < 0.01 as compared to control.

**Table 2:** Ex vivo coagulation time of VRT.

Sample	Dose	aPTT (s)	PT (s)	PT (INR)
Control	saline	28.1 ± 0.5	13.1 ± 0.3	1.00
VRT	0.03 mg/kg	28.6 ± 0.6	13.2 ± 0.4	1.02
	0.06 mg/kg	28.9 ± 0.7	13.3 ± 0.5	1.03
	0.09 mg/kg	29.1 ± 0.6	13.4 ± 0.7	1.05
	0.15 mg/kg	34.7 ± 0.8 *	21.9 ± 0.6 *	3.10 *
	0.3 mg/kg	39.1 ± 0.9 *	26.9 ± 1.0 *	4.87 *
	0.6 mg/kg	45.7 ± 0.7 *	29.9 ± 0.9 *	6.14 *

Each value represents the means ± SD (*n* = 5). * *p* < 0.01 as compared to control.

## Data Availability

The data that support the findings of this study are available from the Corresponding Author (J.-S.B.) upon reasonable request.

## Abbreviations

The following abbreviations are used in this manuscript:

APTT	Activated partial thromboplastin time
DMSO	Dimethyl sulfoxide
ELISA	Enzyme-linked immunosorbent assay
FXa	Activated factor X
HUVEC	Human umbilical vein endothelial cell
MTT	3-(4,5-dimethylthiazol-2-yl)-2,5-diphenyltetrazolium bromide
PRP	Platelet-rich plasma
PT	Prothrombin time
t-PA	tissue-type plasminogen activator
VRT	Veratramine
